# PreVISE: an efficient virtual reality system for SEEG surgical planning

**DOI:** 10.1007/s10055-024-01088-8

**Published:** 2024-12-26

**Authors:** Pascal Spiegler, Haitham Abdelsalam, Owen Hellum, Aristides Hadjinicolaou, Alexander G. Weil, Yiming Xiao

**Affiliations:** 1 Department of Computer Science and Software Engineering, Concordia University, Montreal, Québec Canada; 2 Department of Pediatrics, Division of Neurology, Sainte-Justine University Hospital Center, Montreal, Québec Canada; 3 Department of Surgery, Division of Neurosurgery, Sainte-Justine University Hospital Center, Montreal, Québec Canada; 4 Department of Surgery, University of Montreal Hospital Center (CHUM), Montreal, Québec Canada

**Keywords:** Virtual reality, Neurosurgical planning, Stereoelectroencephalography, Image-guided-surgery

## Abstract

Epilepsy is a neurological disorder characterized by recurring seizures that can cause a wide range of symptoms. Stereo-electroencephalography (SEEG) is a diagnostic procedure where multiple electrodes are stereotactically implanted within predefined brain regions to identify the seizure onset zone, which needs to be surgically removed or disconnected to achieve remission of focal epilepsy. This procedure is complex and challenging due to two main reasons. First, as electrode placement requires good accuracy in desired brain regions, excellent knowledge and understanding of the 3D brain anatomy is required. Second, as typically multiple SEEG electrodes need to be implanted, the positioning of intracerebral electrodes must avoid critical structures (e.g., blood vessels) to ensure patient safety. Traditional SEEG surgical planning relies on 2D display of multi-contrast volumetric medical imaging data, and places a high cognitive demand for surgeons’ spatial understanding, resulting in potentially sub-optimal surgical plans and extensive planning time (~ 15 min per electrode). In contrast, virtual reality (VR) presents an intuitive and immersive approach that can offer more intuitive visualization of 3D data as well as potentially enhanced efficiency for neurosurgical planning. Unfortunately, existing VR systems for SEEG surgery only focus on the visualization of post-surgical scans to confirm electrode placement. To address the need, we introduce the first VR system for SEEG planning that integrates user-friendly and efficient visualization and interaction strategies while providing real-time feedback metrics, including distances to nearest blood vessels, angles of insertion, and the overall surgical quality scores. The system reduces the surgical planning time by 91%.

## Introduction

Epilepsy affects over 70 million people worldwide, with approximately one-third of patients refractory to pharmacotherapy (Löscher et al. 2020). For these patients, surgical intervention that resects or disconnects the brain’s epileptic zone (EZ) has proven to be a highly effective course of action (Chassoux et al. 2018). Stereoelectroencephalography (SEEG) is an invasive technique that is critical for identifying the epileptic zone, where intracerebral electrodes are placed to monitor brain activity (Chassoux et al. 2018). However, intracranial hemorrhage, the main risk of SEEG implantation procedures, occurs in 19.1% of cases (McGovern et al. 2019), potentially resulting in neurological deficits or death (McGovern et al. 2019). Therefore, it is crucial that the implanted intracerebral electrodes avoid critical brain structures, such as blood vessels, without crossing other electrodes. SEEG surgical planning requires multi-contrast/modal volumetric scans to provide key anatomical information such as brain parcellations and vasculatures (Yao et al. 2023). However, traditional planning methods predominantly utilize slice-by-slice 2D display of 3D data in neuronavigation software (Chassoux et al. 2018) by scrolling through the three cardinal cutting planes. Additionally, the user often needs to toggle between different imaging data to inspect various anatomical information (e.g., blood vessels from angiography and surgical targets in T1w MRI). A surgical trajectory is made in a trial-and-error manner until safety constraints have been met by using a mouse to tag the endpoint and selecting the entry point on MRI cutting planes. This can complicate precise 3D anatomical navigation and spatial planning of the SEEG electrodes for the surgeons. Thus, such 2D approaches can be prone to sub-optimal surgical plans, resulting in surgical complications or electrode misplacement for the target brain recording region. Furthermore, due to their cumbersome visualization and interaction strategy, conventional methods also often lead to a prolonged planning time of 2–3 h per patient (~ 15 min per electrode) (Nowell et al. 2016). This highlights the necessity for enhanced and more efficient SEEG surgical planning methodologies.

More recently, data-driven surgical planning tools have been proposed to improve the efficiency and accuracy of SEEG surgical planning. These tools (Pantovic and Essert 2024; Vakharia et al. 2019; De Momi et al. 2014) intend to perform fast and automated electrode trajectory planning by iteratively optimizing a cost function that is made of multiple criteria, such as the trajectory distances to blood vessels and the electrode insertion angles. However, these tools often require manual ground truths (i.e., existing quality surgical plans) to help train the weights of different criteria in the relevant optimization algorithms. Additionally, these weights still may need to be customized for individual cases in a trial-and-error manner. Despite the demonstrated advantages of these tools in the research setting, they are designated as “clinical decision support software” (Vakharia et al. 2019). As per the FDA, when using these tools, “it is not the intent that the healthcare providers rely primarily on any of such recommendations to make a clinical diagnosis or treatment decision regarding an individual patient” (Food and Drug 2022). As a result, to ensure the quality of surgical plans, interactive inspection and correction are still imperative for computer-assisted neuronavigation tools, and may lead to superior quality than fully-automated approaches.

With increasing accessibility, virtual reality (VR) technology can allow for more natural visualization and interaction of complex 3D anatomical data, and has been demonstrated to effectively facilitate medical education and training (Zhao et al. 2020; Tene et al. 2024; Liu et al. 2023). Furthermore, manual SEEG surgical planning in virtual reality can overcome the limitations associated with traditional and automated trajectory planning tools. First, VR surgical planning can streamline the planning process by enabling surgeons to interact intuitively with digital twins of patients’ brains, aiding in the precise mapping of trajectories with a clear spatial understanding of vital structures like blood vessels. Second, it may mitigate dependence on algorithm-generated solutions by fostering the exploration of multiple scenarios and supporting informed decision-making. Third, such a system not only allows surgeons to evaluate their surgical plans using relevant, real-time feedback for key risk metrics, such as distances to blood vessels and angles of insertion, but also offers the capability for easy post-hoc adjustment. Overall, with good design, VR can offer a dynamic and interactive surgical planning method that may enhance the precision and safety of conventional practices, while also reducing the potential bias inherent in fully automated trajectory planning tools due to training data. While various VR-based visualization tools for anatomical data and post-surgical plans have been reported (Phan et al. 2022; Pinter et al. 2020), very few VR systems have been developed to aid in pre-operative trajectory planning. Among the limited studies, Hellum et al. (2022) proposed a VR-based system for efficient Deep Brain Stimulation (DBS) trajectory planning using novel user interaction strategies, including voodoo doll annotation and an eye-tracking-based trajectory design. Their study demonstrated a significant reduction in neurosurgical planning time with high accuracy, showcasing the excellent potential of VR technologies to enhance complex neurosurgical trajectory planning. However, SEEG planning is different from DBS planning: rather than focusing on a single electrode for one surgical target, SEEG electrodes often need to reach two different brain functional regions (i.e., deep and superficial targets/brain regions) and multiple electrodes need to be implanted. This demands customized VR methodologies for robust surgical planning. However, to the best of our knowledge, there are still no known VR systems dedicated to SEEG surgical planning, indicating a significant area for development and research.

In this paper, we present PreVISE (Precision Virtual Integration System for Epilepsy SEEG), the first interactive VR system designed for efficient and robust SEEG surgical planning. We devised three major designs specifically tailored to the clinical task, offering novel contributions to the domain of immersive medical visualization and interaction. First, we employed a dual-brain paradigm to visualize task-specific anatomical information, minimize visual clutter, and remove the need for frequent transparency adjustment of multiple anatomical structures. While one brain model is used to visualize and select surgical targets, the other specializes in trajectory planning. Second, we designed an easy point-drag-pivoting mechanism to allow precise and steady control of the surgical trajectory selection. Lastly, we incorporate real-time feedback metrics, such as trajectory distances to the closest blood vessels, insertion angles, and surgical quality scores to interactively facilitate manual SEEG trajectory design through visual cues, while improving quality and efficiency. By providing surgeons with interactive tools for planning, PreVISE streamlines the process, enables informed decision-making, and allows the evaluation and post-hoc adjustment of surgical plans, while addressing the aforementioned issues in existing SEEG surgical planning tools.

## Materials and methods

In PreVISE, we established an immersive virtual environment with intuitive visualization, interactive real-time feedback, and efficient workflow for the user to perform the clinical task easily and quickly. Its design components are introduced in this section.

### Image processing and anatomical model generation

**Fig. 1:**
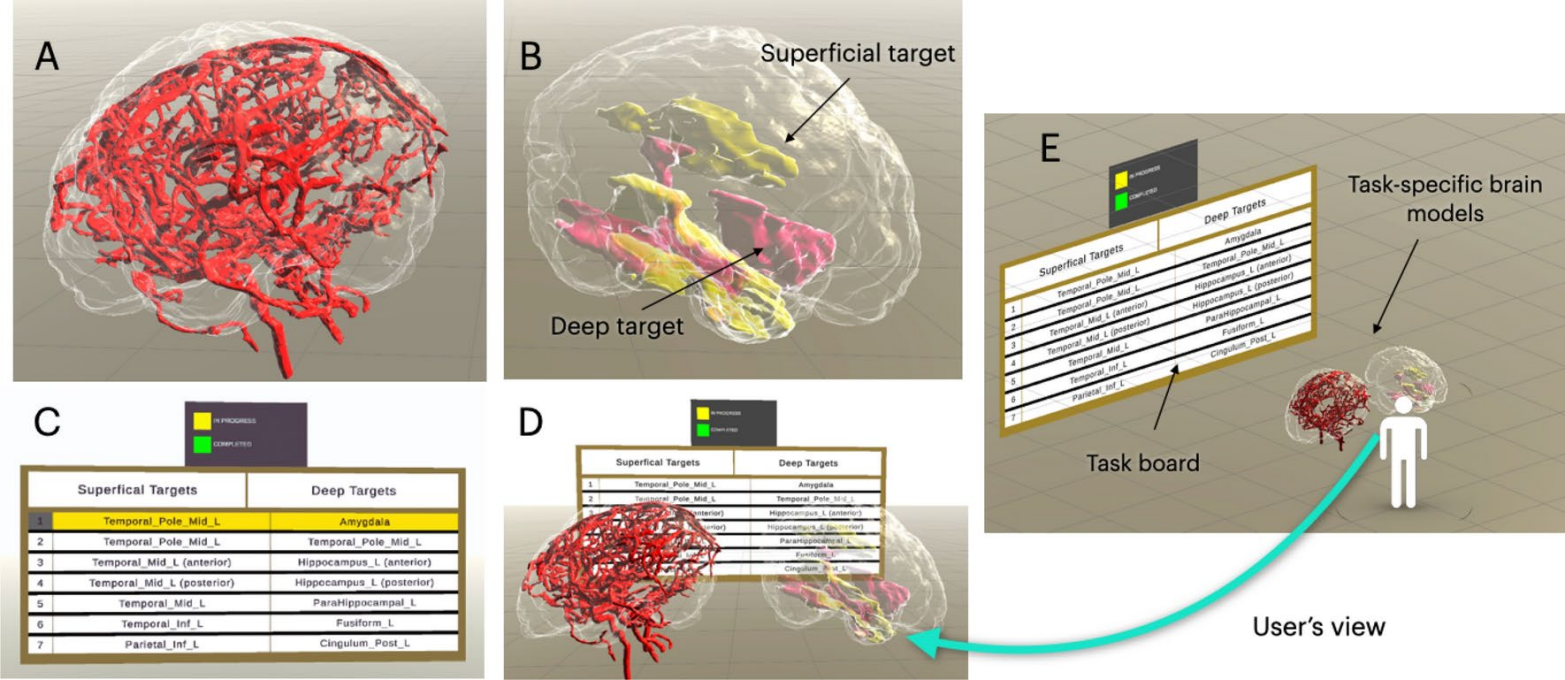
Overview of the main elements in the VR environment. **A** Blood vessel model; **B** brain parcellation model with surgical targets shown for all electrode trajectories (superficial targets = yellow and deep targets = pink); **C** task board showing the list of trajectories, with the first row highlighted to indicate the current task; **D** view of the VR environment from the first person perspective; **E** overview of the user’s placement within the environment

We constructed the virtual brain model (see Fig. 1) for our study with segmentation of 3T MRI scans of a healthy subject’s brain. Upon informed consent, the subject was scanned with T1-weighted MRI ($1 \times 1 \times 1\; \hbox {mm}^{3}$ resolution) and time-of-flight (TOF) MR angiography (MRA, $0.47 \times 0.47 \times 0.70\; \hbox {mm}^{3}$ resolution) using a GE Discovery MR750 MRI scanner. Brain parcellations, brain surface, and cerebral vasculatures were segmented from the MRIs. Specifically, we extracted a brain mask with the BEaST algorithm (Eskildsen et al. 2012) from the T1w MRI. The brain vasculatures were segmented using the Frangi vesselness filter (Frangi et al. 1998) and then further refined manually by the senior author using ITK-SNAP (http://itksnap.org). The detailed 116 brain parcellations (candidates for SEEG targets) were obtained by warping the Automated Anatomical Labelling (AAL)-116 brain atlas (Tzourio-Mazoyer et al. 2002) to the T1w MRI of the subject. Lastly, all segmentations were saved as polygon mesh models in.obj formats to build the 3D scene.

### VR User interface and workflow

#### System setup and VR environment construction

Our VR system for SEEG planning was developed using an Oculus Quest 3 headset and the SteamVR runtime environment. To build the software, the Unity game engine (Version 2021.3.3) was used, and a Razer Blade 15 laptop (Intel Core i7 CPU, NVIDIA GeForce RTX 2070 GPU, and 16.0 GB RAM) was employed for system development and user studies.

As shown in Fig. 1, our VR environment includes two major components. First, a task board with the list of intended electrode positions is displayed in front of the user (Fig. 1C). For each electrode, the deep target, which the end of the electrode must reach, and the superficial target, which the electrode must pass, are shown. The task board is used to guide the current electrode planning task and show the progress of task completion. Second, between the task board and the user, a pair of enlarged virtual brain models are displayed in a mirrored manner (Fig. 1D) with their sides facing the user, both representing the same left hemisphere of the brain. If surgical planning is required also for the right hemisphere, the system allows the left-right flipping of the models to ensure the correct hemisphere is always facing the user. The brain model to the right of the user contains the deep target that the user will select as the endpoint of the trajectory, as well as the corresponding superficial target, both of which are listed on the task board for reference. On the left, the other brain model displays the vasculature of the brain, the endpoint of the electrode trajectory previously selected by the user, and the superficial target. The left brain model is used to design and display the SEEG trajectory while avoiding blood vessels. To facilitate anatomical navigation, semi-transparent brain surface models are visualized for both brain models, with the deep and superficial targets color-coded in pink and yellow, respectively. The targets within these brain models will be updated as the task board progresses for each electrode configuration. The two brain models are enlarged to allow better precision control, and they are fixed in space at a 120° angle for easy reach (Fig. 1E). During software deployment, we observed no lagging or frame freezing.

#### User interaction design and system workflow

**Fig. 2:**
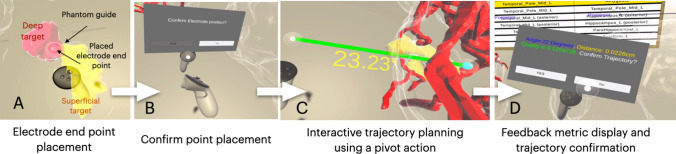
Overview of interaction in the VR environment: **A** Placing an electrode endpoint in the deep target by following the reference point (“phantom guide”); **B** confirming selection; **C** Interactive trajectory planning using a pivot action and real-time risk feedback; **D** surgical plan feedback display and trajectory confirmation

The VR user interface of PreVISE and the system workflow are designed to provide neurosurgeons with a seamless and intuitive experience, aiding them in planning trajectories with precision and efficiency. Upon launching the application on the VR headset, the user is greeted with a tutorial image, which explains the basic operation of the controllers for the designated tasks. To ensure operation consistency, *all operations are performed using the right controller*. Upon clicking the “start” button, the user is introduced to the main VR environment with the task board and two mirrored brain models as described in the previous section. The task board is a crucial element of the user interface (UI) that communicates the key clinical task at hand and the progress of task completion. As a visual guidance, in the task board, the current trajectory underway is highlighted in yellow while the completed trajectories are marked in green. For each SEEG electrode trajectory, the procedure is divided into two main stages: (1) electrode endpoint placement and (2) safe trajectory design. We illustrate the complete workflow in Fig. 2 and as a video demonstration in the supplementary materials.

In the first stage (see Fig. 2A), the user will focus on the virtual brain model on the right-hand-side, which presents the deep and superficial targets as semi-transparent mesh models that correspond to the brain parcellations listed on the task board. To mark the endpoint of the electrode trajectory, the user will need to place a small white sphere attached to the tip of the right controller into the designated location within the deep target structure. This is done by reaching the controller inside the mesh model and pressing the “A” button. To facilitate the user study with non-clinician participants and standardize the validation across different participant groups (i.e., non-clinician vs. clinician), instead of allowing the user to pick the endpoint freely, we provided “phantom guide” points, which were pre-determined by an experienced neurosurgeon specialized in the SEEG procedure. These semi-transparent phantom guide spheres have a VR world-scale (not patient-scale) diameter of 42 mm, chosen to provide a visual cue that indicates the general target area, without overly constraining the participants’ ability to place the 12 mm-diameter world-scale endpoints. Note that in real clinical deployment, we will remove the guidance from the system. Once an endpoint is placed, the left controller vibrates to alert the appearance of a menu that is attached to the same controller. It requests the user to confirm or reject the point placement by using the right controller’s laser pointer and trigger button to click on the associated buttons on the message board. If the point placement is rejected, the procedure will be repeated until satisfaction. Once a satisfactory endpoint placement is confirmed, it will be coded in cyan, and all the endpoints from the previous trajectories will be shown in orange. At the same time, the user will proceed to the second stage.

In the second stage (see Fig. 2C), the user will move to the brain model on the left-hand-side, which contains the blood vessels and the superficial target rendered as a semi-transparent mesh model. Upon confirming the endpoint of the trajectory in the first stage, the position of the point will be mirrored to the vascular brain model and displayed as a cyan-colored sphere. To form a trajectory, the user can reach the right controller close to the endpoint, and click the right trigger button to activate a straight line that connects the endpoint and the tip of the controller. Then, the user will drag the controller outside the brain surface and pivot the straight line around the endpoint to determine a safe trajectory. To facilitate quick decision-making, real-time feedback is provided regarding the angle of insertion with respect to the normal of the brain surface and whether sufficient distance ($\ge$ 2 mm) (Hellum et al. 2022) is ensured from the nearest blood vessel. Specifically, along with the pivoting motion, the angle of insertion is displayed next to the trajectory with red and yellow fonts indicating a value above and below the recommended 30° threshold (Vakharia et al. 2019), respectively. In addition, the color of the trajectory changes based on the distance to the nearest vessels, with red indicating invalid proximity and green indicating valid proximity. Once the user finds a valid trajectory that passes through the designated superficial target, they can press the right controller’s trigger button to save it. Then, similar to the first stage, vibration of the left controller is activated to alert the appearance of a menu displaying the detailed feedback metrics, the surgical quality score (more detail in Sect. 2.2.3), and a request for the user to confirm or reject the planned trajectory. Upon rejection, the user will repeat the planning step until satisfaction. If the trajectory is accepted, then the task board will move to the next trajectory, and the corresponding deep and superficial targets will be updated in the brain models accordingly.

The surgical planning will be conducted for all trajectories until completion, and an averaged surgical quality score, which is the mean of the surgical quality scores from all completed trajectories will be shown to the user. Note that as the brain models were obtained from the MRI scans, the mapping between the MRI scans’ coordinates and those of the virtual environment is available to easily transfer the surgical plan to existing neuronavigation systems.

#### Surgical plan quality metric

Drawing from established research (Bériault et al. 2012; Zelmann et al. 2015), we developed a surgical quality score based on risk factors including the electrode’s distance from the nearest blood vessels and the angle of insertion. This score is in the range [0,1], with a higher value being favored.

To obtain the minimum distance of trajectories from the nearest blood vessels, the relevant distances from the blood vessel mesh model were calculated by checking key points along the determined trajectory. Each key point’s shortest distances (measured in mm) to nearby structures were calculated, and then compared to determine the overall closest vertex on the blood vessel model to the trajectory. A hash table programming structure was used to improve the performance of the algorithm by only checking immediately adjacent vertices of the vascular model for each key point. For the current implementation, 125 points were used to balance accuracy and processing time, with the option to increase points for greater accuracy at the cost of speed.

To evaluate the minimum distance of trajectories from the nearest blood vessels, we implement a score function, $f_d$. Here, distances *d* less than 2 mm are rejected to ensure patient safety from potential hemorrhage (Bériault et al. 2012). Distances within the 2 mm to 5 mm range are scaled accordingly, acknowledging increased risks closer to the 2 mm threshold. For distances beyond 5 mm, where risk reduction plateaus (Zelmann et al. 2015), we assign a maximum score of 1. Therefore:

1 $$\begin{aligned} f_d = {\left\{ \begin{array}{ll} \text {reject} & \quad d < 2\,\text {mm} \\ \frac{d - 2}{5 - 2} & \quad 5\,\text {mm} \ge d \ge 2\,\text {mm} \\ 1 & \quad d > 5\,\text {mm} \end{array}\right. } \end{aligned}$$

Similarly, the angle of insertion is critical for the feasibility and safety of electrode placement. Drawing on the threshold utilized by existing automated planning tools, we define an acceptable angle of insertion, ${\alpha }$, as any angle up to 30° to the normal of the brain surface (De Momi et al. 2014). Angles greater than this threshold may be rejected due to an increased likelihood of complications during keyhole drilling. The angle of insertion score function, $f_{\alpha }$, is therefore given by:

2 $$\begin{aligned} f_{\alpha } = {\left\{ \begin{array}{ll} \text {reject} & \quad \alpha > 30^\circ \\ \frac{30 - \alpha }{30} & \quad \alpha \le 30^\circ \end{array}\right. } \end{aligned}$$

The final surgical quality score, *f*, balances these considerations. As intra-operative hemorrhage is more concerning, following the existing literature (De Momi et al. 2014; Scorza et al. 2017), we assigned higher weight towards the minimum distance to the blood vessel. Thus, the overall score *f*, is defined as follows:

3 $$\begin{aligned} f = 0.8 f_d + 0.2 f_{\alpha } \end{aligned}$$

It is important to note that this metric will only reach 1 if the angle of insertion is 0° and the trajectory is 5 mm or further from blood vessels, which is often not feasible. Therefore, users may maximize this score by first prioritizing maximizing the trajectory’s minimum distance to blood vessels, and then the angle of insertion.

### Experimental design and data analysis

In this section, we describe the methodology and design of our user study, which aims to evaluate the proposed PreVISE system on the factors of system usability, perceived workload, efficiency, and surgical plan quality.

#### Participant recruitment

Upon informed consent, we recruited a total of 17 participants (age = 32.7 ± 6.7 yo, 5 females). To properly assess the proposed system, we balanced the participants with 9 clinician participants from local hospitals (5 neurologists and 4 neurosurgical residents) and 8 non-clinician participants (computer science graduate students). All clinicians actively conduct work and/or research on the topic of epilepsy. To better understand the impact of their background experience with VR technology, neuroanatomy, and SEEG on system evaluation, we asked all participants to rate their familiarity with these three topics on a scale of 1–5, with 5 being the most familiar and 1 being the least. All participants were right-handed and not colorblind. The full demographic information and the background knowledge assessments are summarized in Table 1 (as mean ± std).

**Table 1:** Demographics and familiarity ratings (with VR, anatomy, and SEEG) across different groups (mean ± std)

Group	Demographics	VR familiarity	Anatomy familiarity	SEEG familiarity
All	Sex = 5F & 12 M	3.2 ± 1.5	4.0 ± 1.3	2.5 ± 1.6
	Age = 32.7 ± 6.7 yo			
Non-clinicians	Sex = 2F & 6 M	4.1 ± 1.4	3.4 ± 1.3	1.6 ± 1.2
	Age = 28.8 ± 5.0 yo			
Clinicians	Sex = 3F & 6 M	2.4 ± 1.2	4.6 ± 1.0	3.2 ± 1.6
	Age = 36.1 ± 6.2 yo			
Neurologists	Sex = 3F & 2 M	2.4 ± 1.3	4.2 ± 1.3	2.4 ± 1.1
	Age = 39.8 ± 5.8 yo			
Neurosurgical residents	Sex = 0F & 4 M	2.5 ± 1.3	5.0 ± 0.0	4.3 ± 1.5
	Age = 31.5 ± 2.6 yo			

#### User study design

For the user study, each participant was asked to complete a simulated case of SEEG surgical planning, designed based on the brain model described in Sect. 2.1. In total, seven electrode trajectories were designated, along with their corresponding deep and superficial targets (if applicable) in the left brain hemisphere. The deep and superficial targets were chosen by a senior neurosurgeon, drawing experience from their past surgical cases, and representing common surgical trajectory configurations for SEEG. These configurations are detailed in Table 2. To familiarize the participants with the clinical task and the software, we provided a 3–5-min tutorial using a pre-recorded video of a user completing a single trajectory plan using the PreVISE system. Throughout the video playback, verbal explanations were provided, describing the functionality of the controller interactively. Afterward, the participant would proceed to complete the study with the workflow described in Sect. 2.1. The participants who had eyeglasses were asked to keep them on during the study, and no one reported VR sickness.

**Table 2:** List of simulated SEEG surgical trajectories

	Superfical targets	Deep targets
1	Temporal_Pole_Mid_L	Amygdala_L
2	Temporal_Pole_Mid_L	Temporal_Pole_Mid_L
3	Temporal_Mid_L (anterior)	Hippocampus_L (anterior)
4	Temporal_Mid_L (posterior)	Hippocampus_L (posterior)
5	Temporal_Mid_L	ParaHippocampal_L
6	Temporal_Inf_L	Fusiform_L
7	Parietal_Inf_L	Cingulum_Post_L

The names of the deep and superficial targets correspond to those from the AAL116 brain atlas (Tzourio-Mazoyer et al. 2002). All structures are in the left brain hemisphere

To help evaluate the efficiency and accuracy of the proposed VR system, we recorded quantitative metrics, including the completion time for each electrode trajectory plan, the entry and target positions of the placed electrodes, risk factors for each planned electrode trajectory (minimum distances to blood vessels and insertion angle), and the surgical quality scores (overall, and for each trajectory). Upon finishing the study, the participants were asked to complete a questionnaire including the widely used System Usability Scale (SUS) (Brooke 1995) and NASA Task Load Index (NASA TLX) (Hart 2006) evaluations to assess the usability and perceived workload of the proposed system, respectively. In addition, the participants were also asked to provide freeform comments regarding the positive and negative points of the system, as well as suggestions that can help improve the software design. The mixture of (semi-)quantitative assessments with qualitative feedback can provide a more comprehensive evaluation of the proposed PreVISE system.

#### Quantitative data analysis

From all participants, we collected a range of quantitative data, including the task completion time, electrode endpoint placement, electrode distances to the nearest blood vessels, electrode insertion angles, and surgical quality scores. Additionally, positions of all planned trajectories are saved in JSON format. This allows for the reconstruction and visualization of surgical plans in 3D, as well as the possibility of importing them into other neuronavigational software for further analysis or display purposes. This data is crucial to evaluate the efficacy, efficiency, and accuracy of the proposed system. First, to evaluate the SEEG surgical planning efficiency, we investigate the trajectory design time for each designated electrode and their averaged completion time. Second, as electrode endpoint placement is a crucial element in forming a viable trajectory, we measured the Euclidean distances between the centroids of the “phantom guides” and those of the placed endpoint spheres. In VR, the accurate spatial point placement could be affected by the visualization scheme and user interaction method (Hellum et al. 2023). Low discrepancy between placed endpoints and the phantom guides suggest good reliability in 3D point placement with the proposed visualization scheme, and will ensure accuracy in real clinical deployment when the “phantom guides” are removed. Finally, for the surgical risk factors (minimum trajectory distances to blood vessels and insertion angles), as well as the surgical quality scores, we analyzed the collected values for all trajectories, participants, and sub-groups. To verify the potential differences of the quantitative measurements across different sub-groups, we performed Wilcoxon rank sum tests to compare data between clinicians vs. non-clinicians. For comparisons amongst non-clinicians, neurologists, and neurosurgical residents, one-way analysis of variance (ANOVA) with post-hoc multiple comparison (Tukey) tests were used. Here, a *p*-*value*<0.05 indicates a statistical significance.

#### Semi-quantitative and qualitative data analysis

The System Usability Scale (SUS) evaluation is made of 10 questions on a scale of 1–5, evaluating system complexity, ease of use, and confidence when using a software system. For each of the scores of the 10 questions *x*, odd-numbered questions are scored as $x-1$ while even-numbered questions are scored as $5-x$. Then, all scores are summed and multiplied by 2.5 to produce the final score with the maximum being 100, and a score above 68 indicating good usability (Brooke 2013). On the other hand, the NASA TLX evaluation gauges the perceived workload by considering six factors, including mental demand, physical demand, temporal demand, performance, effort, and frustration levels (Hart 2006), with a rating in the range of 1-21, where a lower score is preferred. To facilitate understanding, the final score for each sub-category of NASA TLX is computed as “(score-1)*5”, resulting in a normalized value range of [0,100]. Lastly, an overall NASA TLX score is obtained as the average of all 6 normalized sub-scores. To examine the group-wise differences regarding perceived workload of the proposed system, we performed Wilcoxon rank sum tests for comparing the NASA TLX scores between clinician vs. non-clinician. For a more nuanced investigation, we used a one-way analysis of variance (ANOVA) and post-hoc multiple comparison (Tukey) tests to reveal the potential differences among non-clinicians, neurologists, and neurosurgical residents. Here, a *p-value* below 0.05 suggests statistically significant differences. Finally, to better understand the potential impacts of demographics (age and sex) as well as personal background knowledge with VR, neuroanatomy, and SEEG surgery on the perceived usability and workload, we also computed their Pearson correlations with the total SUS and overall NASA TLX scores.

For the qualitative data from participants’ freeform questionnaires, we carefully reviewed all the content and summarized the key insights in terms of their frequencies of occurrence among participants. This feedback allows for better understanding of the semi-quantitative assessments and directions for future improvements.

## Results

### Quantitative assessments

#### Planning time

The surgical planning times for each trajectory for all and different participant groups are listed in Table 3. The average planning times per trajectory are 72.6 ± 20.9 s and 80.2 ± 23.9 s for clinicians and non-clinicians, respectively. Of the clinicians, the neurologists had a slower completion time of 75.0 ± 18.1 s/trajectory than the neurosurgical residents (69.6 ± 26.6 s/trajectory). When comparing the time taken for individual trajectories, we found that clinicians had a significantly shorter planning time than non-clinicians for Trajectory 5 targeting *ParaHippocampal_L* and *Temporal_Mid_L* ($p = 0.046$). When analyzing differences between average trajectory planning times, Trajectory 1 took significantly longer than all other trajectory planning times ($p<$ 0.05). However, there were no significant differences among the planning times for Trajectory 2 to 7. Overall, the surgical planning time with PreVISE is reduced by 91% on average compared to traditional methods that take ~ 15 min per trajectory (Nowell et al. 2016). On the other hand, for automated trajectory planning software (Zelmann et al. 2015; Vakharia et al. 2019; De Momi et al. 2014; Nowell et al. 2016), while an initial global optimization typically can take around 1 min or shorter, depending on the algorithms and planning protocols (e.g., iterative refinement), the time for completing a surgical plan can be up to 7 min per electrode.

**Table 3:** Surgical planning time per trajectory across groups (mean ± std, measured in seconds)

Trajectory	Non-clinicians (s)	Clinicians (s)	Neurologists (s)	Neurosurg. res. (s)
1	212.0 ± 107.7	179.6 ± 81.9	172.8 ± 52.6	188.2 ± 118.4
2	77.8 ± 29.9	71.1 ± 20.0	71.3 ± 26.0	70.8 ± 13.0
3	73.2 ± 31.3	68.0 ± 34.1	82.5 ± 39.4	49.8 ± 15.6
4	65.8 ± 42.1	42.7 ± 14.6	40.8 ± 15.9	45.0 ± 14.9
5	53.8 ± 20.7	37.0 ± 19.7	43.3 ± 24.7	29.1 ± 8.1
6	34.1 ± 16.7	58.4 ± 42.7	60.6 ± 52.7	55.8 ± 33.7
7	44.8 ± 17.9	51.2 ± 35.6	53.4 ± 46.0	48.3 ± 23.0
Average	**80.2 ** ± **23.9**	**72.6 ** ± **20.9**	**75.0 ** ± **18.1**	**69.6 ** ± **26.6**

#### Electrode endpoint placement accuracy

The accuracy of electrode endpoint placement was quantified by the Euclidean distance between the centroids of the participant-selected point and the expert-chosen “phantom guide”. As the virtual environment employs enlarged brain models (6 times the size of the real brain) to enhance precision, we report the distances based on *the scale of the virtual model*. Specifically, the clinicians and non-clinical participants achieved mean endpoint placement accuracy of 6.1 ± 0.1 mm and 5.9 ± 0.2 mm, respectively. This translates to ~ 1 mm or 1 voxel in the brain MRI in the patient space. In addition, analyses revealed no significant differences in endpoint placement accuracy between non-clinicians and clinicians, as well as among three sub-groups ($p>$ 0.05).

#### Minimum distance to blood vessels

The mean distance of trajectories from the nearest blood vessels for clinicians was 2.9 ± 0.3 mm, while the non-clinical users recorded a mean distance of 2.8 ± 0.2 mm. Within the clinicians, neurologists had a mean distance of 3.0 ± 0.2 mm, and neurosurgery residents reported a mean of 2.9 ± 0.3 mm. Although no significant group-wise differences were found when considering the averaged value across all trajectories ($p>$ 0.05), when inspecting individual trajectories, Trajectory 6 (targeting the Fusiform_L) revealed a significantly safer/larger margin when planned by the clinicians than the non-clinicians (3.1 ± 0.7 mm vs. 2.4 ± 0.3 mm, *p* = 0.023).

#### Angle of insertion

The average angle of insertion over all trajectories for the clinicians was 20.8° ± 1.6°, compared to 19.5° ± 2.0° for non-clinical users. Neurologists and neurosurgery residents recorded averaged angles of 21.2° ± 1.5° and 20.3° ± 1.7°, respectively, with no significant differences noted among the three sub-groups ($p>0.05$ ). However, when analyzing individual trajectories, a significant difference in insertion angle was observed for Trajectory 6, where the non-clinicians had a significantly lower average insertion angle compared to the clinicians (18.5° ± 1.9° vs. 22.9° ± 3.7°, $p=0.013$).

#### Surgical quality scores

The surgical quality score (see Table 4) helps assess the performance of the user. As expected, averaging over all trajectories, the clinicians’ score (0.293 ± 0.094) was higher than that of non-clinicians (0.273 ± 0.053). Between the neurologists and neurosurgical residents, they achieved scores of 0.302 ± 0.108 and 0.282 ± 0.088, respectively. However, no significant differences were found between clinicians vs. non-clinicians, as well as among the three sub-groups. When examining differences among groups for each trajectory, Trajectory 6 stands out. Here, clinicians had significantly higher scores than non-clinicians (0.329 ± 0.153 vs. 0.182 ± 0.066. *p* = 0.036), due to a higher weight for surgical safety margin in the surgical quality score (as seen in Eq. 3).

**Fig. 3:**
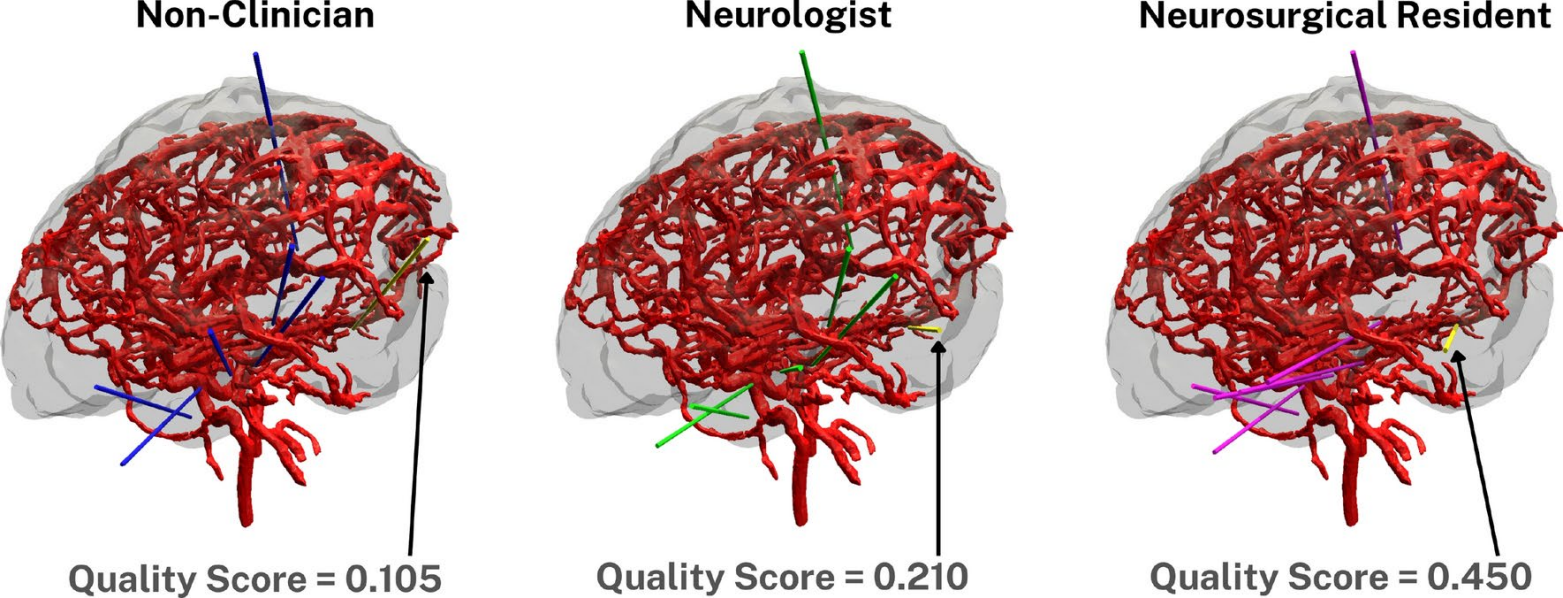
Comparison of electrode placements between a non-clinician, neurologist, and neurosurgical resident. The highlighted yellow trajectories and quality scores represent Trajectory 6 in each case. In the provided examples, the non-clinician achieved a quality score of 0.105 (distance to nearest blood vessel: 2.06mm, insertion angle: 16.7°), the neurologist a score of 0.210 (vessel distance: 2.48 mm, insertion angle: 17.7°), and the neurosurgical resident the highest quality score of 0.450 (vessel distance: 3.56 mm, insertion angle: 24.9°)

To further illustrate the differences in surgical planning between participant groups, we provide a visual comparison of electrode trajectories with Trajectory 6 highlighted in Fig. 3. These visualizations emphasize how clinicians, especially neurosurgical residents, prioritize maintaining a safer distance from critical structures like blood vessels. This approach aligns with the weighting in our surgical quality score formula, where the distance to vasculature has a higher impact on the overall score (Eq. 3).

**Table 4:** Surgical quality scores per trajectory across different groups (mean ± std)

Trajectory	Non-clinicians	Clinicians	Neurologists	Neurosurg. res.
1	0.352 ± 0.121	0.297 ± 0.125	0.290 ± 0.139	0.305 ± 0.125
2	0.205 ± 0.111	0.249 ± 0.088	0.257 ± 0.094	0.239 ± 0.094
3	0.402 ± 0.275	0.350 ± 0.568	0.302 ± 0.747	0.410 ± 0.330
4	0.155 ± 0.083	0.119 ± 0.087	0.149 ± 0.092	0.081 ± 0.074
5	0.433 ± 0.193	0.473 ± 0.201	0.464 ± 0.191	0.484 ± 0.243
6	0.182 ± 0.066	0.329 ± 0.153	0.379 ± 0.161	0.265 ± 0.135
7	0.181 ± 0.066	0.237 ± 0.077	0.273 ± 0.086	0.192 ± 0.036
Overall average	**0.273 ± 0.053**	**0.293 ± 0.094**	**0.302 ± 0.108**	**0.282 ± 0.088**

### Semi-quantitative evaluation

**Fig. 4:**
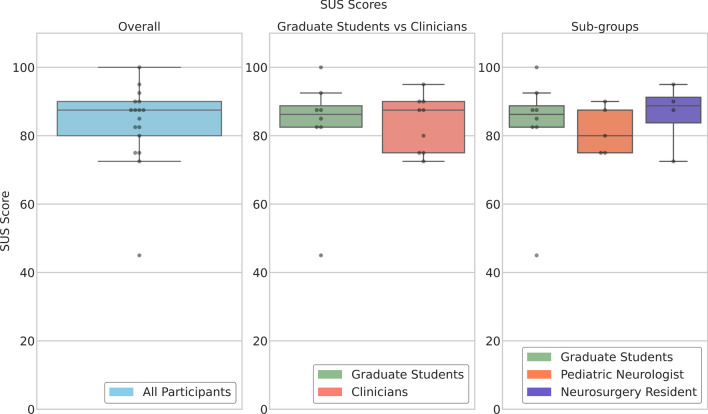
System usability score (SUS) evaluation results

The SUS scores of all participants and different sub-groups are illustrated as boxplots in Fig. 4. The overall SUS score of all participants was 83.2 ± 12.2, which is greater than the threshold value of 68 for a usable system ($p=0.0057$) and is considered an “A” rating (Brooke 1995). Furthermore, the SUS scores were 82.8 ± 16.3 and 83.6 ± 8.1 for the non-clinicians and clinicians. Within the clinicians, neurologists rated PreVISE with a SUS score of 81.5 ± 7.00 while neurosurgical residents rated it at 86.3 ± 9.7. We observed no significant differences among the participant sub-groups in their assessments of system usability ($p> 0.05$).

**Fig. 5:**
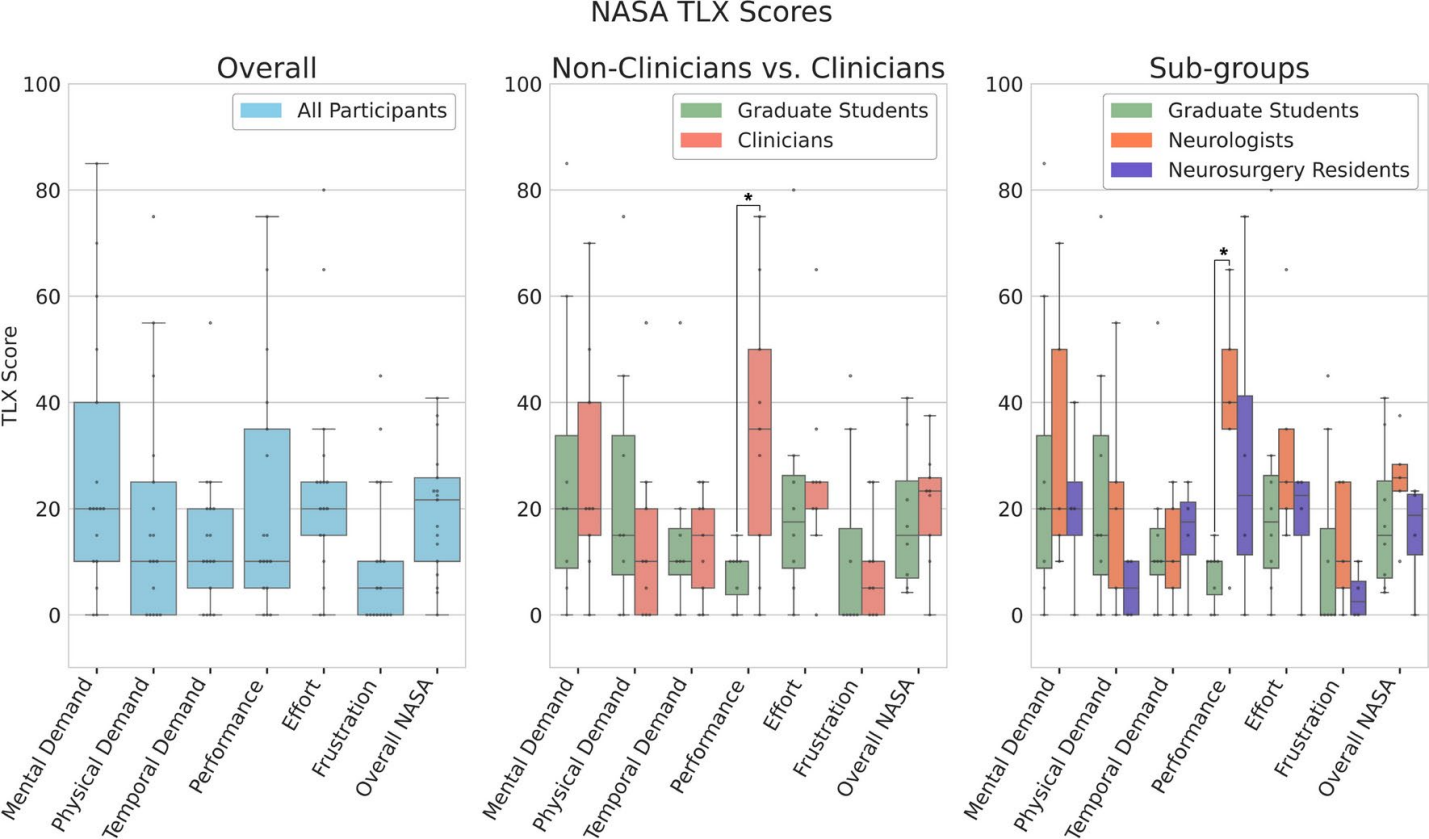
NASA TLX evaluation results

The overall NASA TLX scores of all participants are demonstrated as boxplots in Fig. 5, showing generally low perceived task load when using our system. Specifically, the sub-scores for Mental Demand (27.7 ± 24.9), Physical Demand (18.5 ± 21.7), and Temporal Demand (14.1 ± 13.6) point to a modest level of challenge in both cognitive and physical aspects of the tasks. Scores for Performance and Effort were similarly low (22.1 ± 23.3 and 24.4 ± 20.8, respectively), suggesting general satisfaction with performance and minimal exertion. Frustration had the lowest score (10.0 ± 14.0), indicating very little stress or annoyance. The combined overall score was low (19.5 ± 12.1), suggesting a comfortable but noticeable overall perceived task load.

When comparing NASA TLX scores between non-clinicians and clinicians (Fig. 5), there were no significant between-group differences in the sub-scores, except for the Performance category ($p=0.0125$), where non-clinicians reported lower (better) scores. However, this is in contrast to their lower average overall surgical quality scores than the clinicians (Table 4). Similarly, when comparing the NASA TLX scores among all participant sub-groups (non-clinical users, neurologists, and neurosurgical residents), as shown in Fig. 5, only the Performance category was shown to have significantly lower (better) scores from the non-clinicians than the neurologists ($p=0.040$), but not the neurosurgical residents ($p=0.174$).

Finally, we computed the Pearson correlations of the SUS and NASA TLX scores with the user demographics (age, sex) and the ratings of participants’ familiarity with VR, neuroanatomy, and SEEG. This can help us gain more insights into the impact of the potential factors on the perceived usability and task load. Overall, we found no significant correlations, indicating the robustness of the system across users with various levels of expertise and experience. In terms of the magnitude of the correlations, we found interesting trends. Specifically, the most notable moderate correlations were SEEG familiarity with Physical Demand ($r=-0.396$, $p=0.12$) and neuroanatomy familiarity with Physical Demand ($r=-0.350$, *p*=0.17), potentially suggesting optimization of physical movement related to domain knowledge when using the proposed VR system. Furthermore, a moderate but insignificant positive correlation between SEEG familiarity and system usability was determined ($r=0.25$, $p=0.34$), indicating a possible appreciation of the system’s usability by participants familiar with the operation. This corroborates with our findings, where the neurosurgical residents rated the system with an average usability score of 86.25, the highest among the user sub-groups. Finally, there was a moderate correlation between age and frustration when using the system ($r=0.37$, $p=0.14$), potentially suggesting slower adaptation to the proposed software system with older age. We will further investigate these different aspects in an extended study in the future.

### Qualitative user survey

Besides the quantitative and semi-quantitative metrics, participants were also asked to freely comment on the strengths and weaknesses of PreVISE, as well as offer any suggestions for improvement. Overall, the participants expressed positive opinions towards the proposed system, including praising the user-friendliness of the system (11/17 participants) and the helpfulness of real-time visual feedback such as angle visualization, color responsiveness, and changes in the progress board (5/17 participants). In addition, the participants also shared their critiques and suggestions for improvement of the VR system. Specifically, 3 out of 17 participants mentioned difficulty in visualizing certain targets (e.g., superficial and deep targets) and suggested a need for a different color scheme. Furthermore, 4 participants expressed confusion related to the system’s controller button configurations (pressing A to select electrode endpoints and the trigger for other actions) and would prefer having a single button for all selections.

When specifically looking into the non-clinician group, while most liked the system’s intuitive nature (6/8 participants), half of the group mentioned a desire for additional instructional support, such as in-application prompts and/or additional tutorials to address their lack of knowledge regarding the surgical procedure. In addition, 2 out of 8 appreciated the responsive feedback mechanisms, such as the visual and controller vibration cues, which helped in understanding the system’s functionality.

Among the clinician group, 5 out of 9 participants explicitly commented on the system’s ease of use, and 5 out of 9 mentioned that they found the visualization of the neuroanatomy appealing, which is an important dimension for VR software systems. Notably, all neurosurgical residents suggested visualizing additional anatomical and physiological information, including detailed sulci patterns and imaging modalities related to brain connectivity (e.g., white matter tractography and functional MRI), which could further facilitate decision-making in SEEG surgical planning. Finally, the neurosurgical residents, who were most familiar with SEEG surgery, stated that the “3D visualization allows faster trajectory planning” and that “the automated angle calculation is useful since [it is] hard to figure out when planning in 2D”. It was also acknowledged by a neurosurgical resident that the planning speed when using PreVISE is significantly faster than using conventional neuronavigational SEEG planning software, confirming our quantitative finding of a 91% reduction in average trajectory planning time.

## Discussion

To allow efficient and robust SEEG surgical planning, we designed a novel workflow with tailored data visualization and interaction strategies. In conventional surgical navigation systems, the user often needs to toggle between different medical scans and image segmentation while frequently adjusting their transparency levels. In a VR environment, this can greatly hinder efficiency while complicating the user interface, due to the requirement of additional functional buttons and/or physical actions. In our PreVISE system, we proposed to visualize two task-specific virtual brain models to minimize the need for frequent visual element adjustments. In addition, instead of allowing the user to manipulate the brain model’s positioning and orientation freely, we fixed the model in space to encourage active exploration and simplify the operations. To help quickly design a viable surgical plan, we designed a pivot action with real-time surgical risk feedback using visual cues. Previously, to enable speedy electrode trajectory design, Hellum et al. (2022) proposed to leverage the line-of-sight obtained by eye-tracking from the VR headset. Different from deep brain stimulation surgery, SEEG electrode placement often needs to consider both deep and superficial targets. The occlusion of the electrode endpoint by this superficial target can pose challenges using line-of-sight for SEEG trajectory planning, which is why the interaction paradigm was excluded from this study (besides the fact that currently eye-tracking is only available in high-end VR headsets). As many automatic SEEG trajectory planning tools may require further manual adjustment, in addition to being used as a standalone tool, our PreVISE system can also be employed to provide further intuitive surgical planning refinement for automatic planning software.

In the semi-quantitative evaluation, PreVISE reached a high SUS score of 83.2 ± 12.2. This echoes the qualitative feedback from the participants, where the majority of them mentioned that the system was easy to learn and intuitive. Furthermore, the average NASA TLX score of 19.5 ± 12.1 suggests a light but noticeable workload. This is understandable for a specialized clinical task. The NASA TLX score corresponds with the comments received during interviews, where participants mentioned that PreVISE was not tiring, but still required some effort, particularly with the functional button configurations of the controllers, and the non-clinicians’ demand for an additional tutorial on the clinical knowledge. To introduce the software to the participants, we used a short first-person perspective video recording while explaining the functionality of the controllers interactively. For the first electrode trajectory, participants often required additional time to become familiar with the software, making its planning time less representative of the true potential of the proposed system. However, the first trajectory planning is still under 4 min on average, which is shorter than some automated planning software [e.g., 7 min/electrode in Zelmann et al. (2015)], and for the rest of the trajectories, the speed of task completion is much faster (~ 1 min). We will devise a hands-on tutorial with a short task-specific practice, which was successfully adopted in a single-trajectory planning VR system (Hellum et al. 2022) for our system to mitigate this. Noted in our freeform questionnaire, some participants mentioned the inconsistency in controller button configuration for object selection and the need for alternative color schemes for anatomical models. For our future system improvement, we will use the trigger button for all object selection to ensure consistency while adding an additional menu to allow the change of mesh model colors.

The participants’ performance, which we measured quantitatively through minimum distances to blood vessels, angles of insertion, surgical quality scores, and electrode endpoint placement accuracy, was similar across all groups. For each of these metrics, when averaged across all trajectories, there was no significant difference between the performance of clinicians and non-clinicians. However, this was not always the case for individual, more difficult trajectories. For example, for Trajectory 6, which involves the left fusiform gyrus (deep target) and left inferior temporal cortex (superficial target), there is more complex vasculature in the region and the brain surface curvature (near the bottom edge of the brain) is steeper, making it slightly more challenging than the other trajectories. Therefore, the clinicians performed significantly better than the non-clinicians, with a significantly safer distance from blood vessels. We also observed that for this trajectory, the non-clinicians incorrectly prioritized the angle of insertion more than the distance, achieving a significantly lower angle of insertion. This is likely because clinicians are aware that the risks of intra-operative hemorrhage are more concerning (De Momi et al. 2014; Scorza et al. 2017) than the angle of insertion due to their medical backgrounds. While clinicians had higher average surgical quality scores than non-clinicians (0.293 ± 0.094 vs. 0.273 ± 0.053), interestingly, they self-assessed their performance as worse than the non-clinicians. We may speculate that this could be attributed to a combination of overconfidence among the non-clinicians and the medical background of the clinicians contributing to an increased awareness of potential risks. This is especially the case for the neurosurgical residents. These observations highlight the importance of effective training tools to enhance surgical planning skills and risk awareness among both novice and experienced practitioners. Our PreVISE system is well-suited for educating and training surgical residents. The immersive VR environment allows residents to interact with detailed 3D anatomical models, receive real-time feedback through surgical quality metrics, enhance their understanding of neuroanatomy, and improve their skills in electrode trajectory planning. Furthermore, since our system supports display mirroring to an external monitor, neurosurgeons can guide residents by observing their actions in real time while providing verbal guidance and feedback. This feature facilitates a mentor-mentee learning environment, even though the current system is designed for individual use and does not yet support multi-user interaction within the VR environment.

There are a few limitations in our investigation. First, as SEEG electrode implantation is a highly specialized surgical procedure, recruiting a large number of surgeons and surgical residents who are familiar with it for user studies is challenging. As a result, our recruited participants include both clinicians and non-clinicians. This limited fine-grained investigation for between-group analyses on some level, and thus the insights drawn from the statistical analyses would need to be further confirmed with a larger cohort. However, the small discrepancy of different evaluation metrics between clinicians and non-clinicians further confirms the robustness and high usability of our proposed system. Upon further improvement based on the feedback, we will seek to conduct a larger scale study with more clinicians in the future. Second, we had limited access to commercial neuronavigation software (e.g., Medtronic Stealthstation), which has a steep learning curve for the non-clinician participants; we didn’t include a comprehensive comparison between the traditional and proposed VR methods. We will further refine the system based on this preliminary study and conduct more extensive validation that compares traditional and VR-based methods in an extended study. Third, in our preliminary study, we designed the overall surgical plan quality score based on the electrodes’ distances to the nearest blood vessels and insertion angles, which are primary clinical considerations common in the relevant literature to reduce surgical risks and complications. However, so far, there is still a lack of consensus on the criteria for the quality of electrode trajectory planning, and the implementation varies in the literature. We will explore additional metrics with the possibility of involving additional imaging data, such as the coverage of grey matter volume, in future software updates. Additionally, our study focused on unilateral implantation with a moderate number of electrodes, reflecting common clinical practice. While dense or bilateral implantations are less common, with the former generally avoided to minimize complexity and risks, they may pose challenges due to potential visual clutter in the VR environment. In the future, we plan on also exploring dense and bilateral electrode arrangements, ensuring PreVISE is effective across different clinical scenarios. Finally, with the consideration of the limited duration of the user study sessions, we only tested one brain model. With individual anatomical variations, difficulty levels of trajectory planning can also vary. To further ensure the usability of the proposed system, we will incorporate additional patient data in future studies.

As blood vessel segmentation and visualization is not the focus of our work, we used manually revised automatic blood vessel segmentation to ensure the correct inclusion of the full vasculatures from the MR angiography to the best of our capacity in our experiments. More advanced segmentation and rendering algorithms are in rapid progress, and may offer enhanced accuracy for 3D vascular representation. To mitigate the requirement for advanced segmentation techniques, one future add-on for our system can include a 2D medical image viewer to allow further verification of the surgical plan, potentially in a slice-by-slice manner for the angiography scans along the designed trajectory. Here, we employed phantom guides to help participants select the end points, which are typically proposed by the neurologists based on potential hypotheses of seizure locations while the trajectories are planned by the surgeons. In our user study, the neurologists and neurosurgeons performed both tasks. Further considering the limited clinical knowledge in the graduate student group, the use of phantom guides allows direct comparisons of these three groups in a controlled setting to focus on the efficacy of the proposed software system. In practice, variants of the phantom guides could still serve as a prior knowledge to facilitate surgical target selection (Scorza et al. 2020) in the future development of the system.

Although we have demonstrated the high potential of the proposed PreVISE system, a few further considerations could greatly facilitate its clinical adoption in the future. First, like existing automated trajectory planning software (Zelmann et al. 2015; Vakharia et al. 2019; De Momi et al. 2014; Nowell et al. 2016), anatomical segmentation is necessary for our system. With the advancement of deep learning-based medical image segmentation techniques, anatomical mesh model preparation for PreVISE could be shortened to just a few seconds, but a separate module for this will still be required. Second, while the coordinates of the planned trajectories can be saved in the same space as the patient MRIs, cross-platform protocols still need to be developed to allow easy transfer of completed surgical plans to existing neuronavigation systems in the operating room. Third, VR fatigue can be a potential issue for extended use of the system, but could be mitigated with fast workflow and continuous development of VR displays. Lastly, our current system recommends a 2 × 2 m^2^ area for comfortable operation, but this could be further reduced if appropriate virtual “teleportation” action is implemented.

## Conclusion

In conclusion, we have designed and implemented a novel VR-based software, PreVISE, to allow for more intuitive and efficient surgical planning before SEEG electrode implantation. With tailored visualization and interaction strategies, the system is shown to decrease planning times by 91%, and have robust performance as confirmed through user studies with both non-clinicians and clinicians working closely with epilepsy treatment. We will further improve the system based on the participant feedback in the future, and employ additional patient data to fully test the end software system.

## Data Availability

The data that support the findings of this study are available from the corresponding author upon reasonable request.
